# Relation Between Diabetes and Psychiatric Disorders

**DOI:** 10.7759/cureus.30733

**Published:** 2022-10-26

**Authors:** Kishan Akhaury, Sarika Chaware

**Affiliations:** 1 Physiology, Jawaharlal Nehru Medical College, Datta Meghe Institute of Medical Sciences, Wardha, IND; 2 Community Medicine, Jawaharlal Nehru Medical College, Datta Meghe Institute of Medical Sciences, Wardha, IND

**Keywords:** schizophrenia, anxiety, mood disorder, psychiatry, diabetes

## Abstract

Depression, anxiety, and schizophrenia are all things that have been found to be linked to the mental health of diabetics. When combined with particular mental health conditions, the management of diabetes might become very challenging. The management of diabetes requires the patient to be actively involved and is contingent on the patient's adherence to prescribed lifestyle modifications, self-monitoring, and medication. Patients who struggle to keep their mental health concerns under control are more likely to have trouble managing their diabetes on their own. People who have diabetes, because it is one of the most cognitively and behaviorally taxing chronic medical conditions, may be especially susceptible to developing mental health disorders. It is more prevalent in those who suffer from mental problems than it is in the general population. Diabetics frequently struggle with a variety of mental health conditions, including but not limited to schizophrenia, anxiety, and depression. Diagnosing and treating mental health disorders is an important component of diabetes treatment that can be accomplished through the collaborative efforts of members of a multidisciplinary team. Patients who fall into this category can benefit from a wide range of services provided by pharmacists who work with them, including individual assessments, joint goal-setting, skill development, ongoing monitoring, and medication management. These services are designed to help patients feel better and function better.

## Introduction and background

Endocrinologists and psychiatrists have long been interested in the intersections between diabetes and mental health treatment. In the 17th century, Thomas Willis argued that diabetes was caused by "lasting melancholy and various depressions" [1]. Within a decade of its discovery, insulin was being used in a psychiatric treatment known as insulin coma therapy. It is only in the last few decades that this connection has received substantial scientific attention. Diabetes and mental health issues have a two-way relationship, with each condition influencing the other in different ways. Several aspects of this link are covered in this article. Tobacco and alcohol, for example, have been demonstrated to have pharmacokinetic effects on oral hypoglycemic medicines [2]. Furthermore, the presence of a coexisting psychiatric disorder, such as depression, may impede diabetes management by lowering medication adherence [1]. Blood glucose testing and insulin injection are only two examples of how investigations and treatments can be complicated by diseases like needle and injection anxiety. Patients with mental health issues are also less likely to seek help [2].

## Review

Lifestyle factors

It is theorized that lifestyle variables may initiate or exacerbate the association between depression and diabetes. Those who suffer from depression are at a higher risk of acquiring type 2 diabetes, in part because they are less active and more likely to consume a diet high in saturated fats and refined carbohydrates while cutting back on fruit and vegetables [3-5]. Patients with a diabetes diagnosis who are simultaneously suffering from depression symptoms are also more likely to not adhere to self-care management [6,7]. In a meta-analysis of 49 separate samples, noncompliance with diabetes treatment guidelines, such as attending medical visits, eating well, exercising, taking medications as prescribed, checking glucose levels, and caring for one's feet, was substantially related to depression [8,9]. This backs up the idea that not taking care of yourself well can lead to high blood sugar, which can make depressive symptoms worse and make it harder to take care of yourself.

Delirium

Hypoglycemia [10] and diabetic ketoacidosis [11] are two potential causes of delirium in people with diabetes. As far as clinical manifestations go, delirium is the most extreme form of these phases. Hypoglycemia delirium is more common in patients with preexisting mental health conditions [11]. It is impossible to determine the true prevalence of delirium in diabetes with existing noesiological methodologies due to the use of overlapping terminologies in the body of research that is currently available. However, low blood sugar (hypoglycemia) or diabetic ketoacidosis can lead to delirium, and both of these conditions are common in diabetes patients [12,13]. Several unfavorable consequences have been related to delirium, including lengthened hospitalizations, diminished cognitive and functional abilities, increased morbidity, and even death [14,15]. The clinical picture is characterized by reduced psychomotor activity and an overall sense of calm. Also shared by both forms is a sense of confusion and diminished sensory abilities. Hallucinations, altered sleep-wake patterns, and impaired cognitive function are all clinical features of delirium. Usually, there will be bright spots intermingled with cloudy patches. Prompt detection of delirium can considerably improve the prognosis. While palliative care is important, it is secondary to addressing the underlying cause of the patient's suffering. Low doses of dopaminergic antagonists (typical antipsychotics) may be used to control disruptive behavior [12]. Powerful medications like haloperidol are recommended. 

Tobacco

There are combustible (cigarettes, bidi, hookah, and cigars) and noncombustible (chewing tobacco) varieties of tobacco (gutkha, tobacco powder, *khaini*, snuff). It has been shown that the number of smokers among people with diabetes is similar to that seen in the general population, at least in research conducted by Western researchers [16]. One of the risk factors for developing diabetes is cigarette smoking, although it is a risk factor that may be managed. The increased risk of diabetes appears to be dosage-dependent [17,18]. The use of smokeless tobacco has also been related to an increased risk of acquiring type 2 diabetes, albeit the data is less compelling [19]. When a person smokes cigarettes, they increase their chances of developing microvascular complications like nephropathy, retinopathy, and neuropathy, as well as macrovascular issues like coronary heart disease (CHD), stroke, and peripheral vascular disease (the strongest association in type 2 diabetes) [20]. Gutkha chewing is associated with more severe periodontal disease and tooth pain in diabetics [21]. Hyperglycemia, hyperinsulinemia, and hypertension are postulated to be caused by smoking, as are impaired endothelial function and the pro-diabetogenic activity of tobacco smoke components (e.g., cadmium) [22]. Important questions to ask and provide to patients regarding tobacco use include whether or not they smoke. Most young people with diabetes start smoking soon after their diagnosis, so this is very pertinent for this population [23]. Smoking cessation is associated with weight gain and an increased risk of type 2 diabetes, both of which clinicians should be prepared to address [24]. These effects, however, are either temporary or easily managed through behavioral and lifestyle changes [25]. So, in the years after quitting, smokers should get help managing their weight and be checked for diabetes. Also, it is suggested that the doses of different oral hypoglycemic medicines that are processed by the enzyme system be monitored since smoking makes the cytochrome P450 system make different isoforms. The various drugs used for the treatment of tobacco abuse are listed in Table 1.

**Table 1:** The various drugs used for the treatment of tobacco abuse

Drug	Mechanism of action	Side effects/contraindications/recommendations
First-line therapies: nicotine replacement therapy (NRT): available as gums (2 mg/ 4 mg per piece) and patches	Prevents withdrawals associated with abstinence from tobacco products	Mild and transient and include mouth soreness, hiccups, and jaw ache, local skin reaction
Varenicline	Partial agonist at alpha 2, beta 4 nicotinic receptors	Use associated with increased risk of suicidal behavior and cardiovascular events
Bupropion (sustained release)	Blockage of reuptake of dopamine and norepinephrine	Preferred for comorbid depression and/or weight concerns Contraindications: history of seizure disorder, eating disorder; use of a monoamine (MAO) inhibitor in the past 14 days
Second line therapies: Clonidine	Alpha 2 adrenergic receptor agonist	Dry mouth, drowsiness, dizziness, hypotension, and sedation To be used with caution in hypertensive patients specially during induction and withdrawal phases
Nortriptyline	Tricyclic antidepressant	Sedation, dry mouth, blurred vision, urinary retention, lightheadedness, tremors. Overdose may produce cardiotoxic effects

Alcoholic beverages 

Epidemiological studies and populations seeking treatment have shown that alcoholic beverage consumption is prevalent among people with diabetes, with estimates ranging from 50% to 60% [26,27]. Drinking and developing diabetes is a topic of ongoing debate. Moderate consumption (up to one drink a day for women and up to two drinks a day for men. Examples of one drink include beer: 12 fluid ounces (355 milliliters) and wine: 5 fluid ounces (148 milliliters), which has been shown to be healthy, whereas high consumption is associated with an increased risk of type 2 diabetes [28]. Acute pancreatitis brought on by alcohol consumption might bring on glucose intolerance in alcoholics. Alcohol consumption in diabetes is associated with a number of complications, one of the most common and serious of which is the onset of hypoglycemia. It could be fasting hypoglycemia from alcohol use, drug-induced hypoglycemia that is amplified by alcohol consumption, or reactive hypoglycemia in people who are particularly vulnerable to these conditions. In addition, drinking alcohol can impair one's ability to recognize the first signs of a psychotic episode and take preventative measures [26]. Ketoacidosis from diabetes can progress quickly with alcohol consumption. Diabetic peripheral neuropathy and alcoholic retinopathy can both be caused and exacerbated by the use of these substances. According to a study [29], adherence to diabetes self-care activities is inversely connected to alcohol use. There may be a disulfiram-ethanol interaction if you take chlorpropamide (a sulfonylurea medicine) and drink alcohol together. Alcohol can also induce all of these symptoms simultaneously. In addition to this, drinking alcohol has been related to significant weight gain as well as raised blood sugar levels. In addition, the metabolism of oral hypoglycemic medications can be altered by alcohol consumption. Metformin shouldn't be taken by people who drink a lot of alcohol because it could cause them to get lactic acidosis [29]. Oral hypoglycemics are metabolized in the liver, so people with alcohol-induced hepatopathy may need to reduce their dosage. The various drugs used for the treatment are mentioned in Table 2.

**Table 2:** The various drugs used in the treatment of alcohol abuse GABA: gamma-aminobutyric acid

Drug	Mechanism of action	Side effects/contraindications/recommendations
Disulfiram	Irreversible inhibition of enzyme acetaldehyde dehydrogenase (ALDH)	Transient mild drowsiness, fatigue, impotence, headache, acneiform eruptions, allergic dermatitis, and a metallic or garlic-like aftertaste
Acamprosate	Enhancement of GABAergic neurotransmission and reduction of glutamatergic neurotransmission	Transient diarrhea, headaches, dizziness, and pruritus; safe in hepatopathy as not metabolized in liver
Naltrexone	Opioid-receptor antagonism	Nausea, headache, anxiety, sedation, dose-dependent hepatotoxicity
Topiramate	Antagonism of glutamate and facilitation of gamma-aminobutyric acid (GABA)	Dizziness and somnolence, ataxia, impaired concentration, confusion, fatigue, paresthesias, speech difficulties, diplopia, and nausea
Selective serotonin reuptake inhibitors (SSRIs)	Inhibition of serotonin reuptake at pre-synaptic level	Limited evidence for their use

Mood imbalances

Mood disorders include conditions including depression, mania, and hypomania. Multiple epidemiological and clinical research have found a correlation between diabetes and mental health issues [30]. Greater morbidity and death have been associated with this combination. According to a recent study [31,32], patients with diabetes have a 50-100% higher risk of depression than the overall population. Hospital-based research and epidemiological surveys have found that the prevalence of diabetes among bipolar disorder patients is either greater than in the general population or identical [33,34]. Depression due to diabetes is correlated with a higher incidence of complications, mortality, and healthcare costs [35-37]. There is a two-way causal connection between diabetes and depression. It is believed that depression contributes to the onset of diabetes [37]. A recent meta-analysis discovered that people who also suffered from depression had a 60% increased risk of developing diabetes [38]. Research has found that all forms of depression, including mild, chronic, and untreated depression, increase the risk of developing diabetes [39]. Mood stabilizers and antipsychotics used to treat bipolar disorders have a more robust database in regard to metabolic derangements. As the term "phenothiazine diabetes" from the 1960s shows, the connection between antipsychotics and diabetes was recognized early on. Due to their metabolic and cardiovascular side effects, newer atypical antipsychotics have attracted a lot of attention. According to Hermanns et al., people who take these medicines are more likely to gain weight and have a harder time handling glucose [40].

Anxiety problems 

In comparison to the general population, people with diabetes are substantially more likely to experience anxiety issues [41]. Research has found that anxiety disorders and their associated symptoms are strong independent predictors of developing diabetes. According to Green et al., the risk of developing an anxiety illness is inversely associated with hemoglobin A1c levels [42]. Generalized anxiety disorder (GAD) has been proven to have a prevalence that is almost three times higher than that reported in the general population [43]. On the other hand, the frequencies of these disorders were found to be consistent with those reported in population studies: panic disorder, obsessive-compulsive disorder, post-traumatic stress disorder (PTSD), and agoraphobia [44]. In comparison to studies into the relationship between depression and diabetes, research into the relationship between anxiety disorders and diabetes is sparse. Depression and anxiety in the context of diabetes have been studied mostly together. Both hypoglycemia episode phobia and needle and injection phobia have been linked to diabetes [45]. They are more likely to neglect glucose monitoring and insulin dosing. Additionally, prolonged hyperglycemia could be maintained due to anxiety about hypoglycemia. Clinical signs such as sweating, nervousness, trembling, tachycardia, and confusion are similar between hypoglycemic episodes and anxiety disorders. The fear of hypoglycemic episodes makes this a potential diagnostic concern. People who worry a lot may be more likely to misread or ignore the signs of low blood sugar.

Schizophrenia and other psychotic conditions

Diabetes is strongly associated with a number of medical conditions, one of which is psychosis. Compared to other diseases, people who have been diagnosed with schizophrenia are at higher risk to get type 2 diabetes [40]. People who are related to schizophrenia patients, even first cousins, are at a considerably higher risk of developing type 2 diabetes. Because of a favorable family history, people with schizophrenia may have a threefold increased risk of developing diabetes [41]. According to the available data, patients with both diabetes and schizophrenia have an increased risk of death [42]. More people with schizophrenia are diagnosed with type 2 diabetes, which is linked to a higher mortality rate for those who have schizophrenia. Low glucose tolerance and insulin resistance have both been associated with schizophrenia [37]. Schizophrenia patients may have impaired glucose tolerance at a rate as high as 30%, depending on their age. Schizophrenia patients have an increased chance of developing diabetes, which is likely due to both hereditary and environmental causes. Not getting enough exercise, eating poorly, not getting proper medical care, and using antipsychotic drugs are also contributors. Initial studies suggest that schizophrenia may be a separate risk factor for developing diabetes [37,38,44]. Furthermore, there is a 50% nonadherence rate among those with schizophrenia who are receiving treatment. Because of this, their managers will be forced to make some tough decisions.

## Conclusions

Diabetes is a growing public health concern. Many diabetics struggle to improve their diabetes control, often due to mental illnesses or psychological and social issues. Diabetes has serious consequences for the individual and, if not addressed, can lead to complications such as blindness, kidney failure, and even amputations. There are also implications for healthcare services as a result of increased admissions and emergency department presentations with diabetes-related complications. The long-term costs associated with complications such as renal failure and amputation are significant. Addressing the psychiatric and psychological barriers to good glucose control can help reduce the individual and societal burdens of diabetes and its complications.

## References

[1] Goldney RD, Phillips PJ, Fisher LJ, Wilson DH (2004). Diabetes, depression, and quality of life: a population study. Diabetes Care.

[2] Hutter N, Schnurr A, Baumeister H (2010). Healthcare costs in patients with diabetes mellitus and comorbid mental disorders--a systematic review. Diabetologia.

[3] McMartin SE, Jacka FN, Colman I (2013). The association between fruit and vegetable consumption and mental health disorders: evidence from five waves of a national survey of Canadians. Prev Med.

[4] Payne ME, Steck SE, George RR, Steffens DC (2012). Fruit, vegetable, and antioxidant intakes are lower in older adults with depression. J Acad Nutr Diet.

[5] Weyerer S (1992). Physical inactivity and depression in the community. Evidence from the Upper Bavarian Field Study. Int J Sports Med.

[6] Gonzalez JS, Peyrot M, McCarl LA, Collins EM, Serpa L, Mimiaga MJ, Safren SA (2008). Depression and diabetes treatment nonadherence: a meta-analysis. Diabetes Care.

[7] Gonzalez JS, Safren SA, Cagliero E (2007). Depression, self-care, and medication adherence in type 2 diabetes: relationships across the full range of symptom severity. Diabetes Care.

[8] Lewis R Diabetic emergencies: Part 1. Hypoglycaemia. Diabetic emergencies: Part.

[9] Boland E, Monsod T, Delucia M, Brandt CA, Fernando S, Tamborlane WV (2001). Limitations of conventional methods of self-monitoring of blood glucose: lessons learned from 3 days of continuous glucose sensing in pediatric patients with type 1 diabetes. Diabetes Care.

[10] Kitabchi AE, Umpierrez GE, Murphy MB, Barrett EJ, Kreisberg RA, Malone JI, Wall BM (2001). Management of hyperglycemic crises in patients with diabetes. Diabetes Care.

[11] MacLullich AM, Beaglehole A, Hall RJ, Meagher DJ (2009). Delirium and long-term cognitive impairment. Int Rev Psychiatry.

[12] Han JH, Shintani A, Eden S (2010). Delirium in the emergency department: an independent predictor of death within 6 months. Ann Emerg Med.

[13] Ford ES, Mokdad AH, Gregg EW (2004). Trends in cigarette smoking among US adults with diabetes: findings from the Behavioral Risk Factor Surveillance System. Prev Med.

[14] Foy CG, Bell RA, Farmer DF, Goff DC Jr, Wagenknecht LE (2005). Smoking and incidence of diabetes among U.S. adults: findings from the Insulin Resistance Atherosclerosis Study. Diabetes Care.

[15] Willi C, Bodenmann P, Ghali WA, Faris PD, Cornuz J (2007). Active smoking and the risk of type 2 diabetes: a systematic review and meta-analysis. JAMA.

[16] Persson PG, Carlsson S, Svanström L, Ostenson CG, Efendic S, Grill V (2000). Cigarette smoking, oral moist snuff use and glucose intolerance. J Intern Med.

[17] Eliasson B (2003). Cigarette smoking and diabetes. Prog Cardiovasc Dis.

[18] Javed F, Altamash M, Klinge B, Engström PE (2008). Periodontal conditions and oral symptoms in gutka-chewers with and without type 2 diabetes. Acta Odontol Scand.

[19] Schwartz GG, Il'yasova D, Ivanova A (2003). Urinary cadmium, impaired fasting glucose, and diabetes in the NHANES III. Diabetes Care.

[20] Shaw NJ, McClure RJ, Kerr S, Lawton K, Smith CS (1993). Smoking in diabetic teenagers. Diabet Med.

[21] Yeh HC, Duncan BB, Schmidt MI, Wang NY, Brancati FL (2010). Smoking, smoking cessation, and risk for type 2 diabetes mellitus: a cohort study. Ann Intern Med.

[22] Yeh HC, Duncan BB, Schmidt MI, Wang NY, Brancati FL (2010). Smoking, smoking cessation, and risk for type 2 diabetes mellitus: a cohort study. Ann Intern Med.

[23] Lethbridge-Cejku M, Schiller JS, Bernadel L (2004). Summary Health Statistics for US Adults: National Health Interview Survey, 2002. Report no 10. Summary Health Statistics for US Adults: National Health Interview Survey, 2002. Report no 10.

[24] Ahmed AT, Karter AJ, Liu J (2006). Alcohol consumption is inversely associated with adherence to diabetes self-care behaviours. Diabet Med.

[25] Li H, Wang G, Wang A, Tong W, Zhang Y (2011). Alcohol consumption and risk of type 2 diabetes in Mongolian population, inner Mongolia, China. J Diabete Metab.

[26] Emanuele NV, Swade TF, Emanuele MA (1998). Consequences of alcohol use in diabetics. Alcohol Health Res World.

[27] Katon WJ, Lin EH, Williams LH (2010). Comorbid depression is associated with an increased risk of dementia diagnosis in patients with diabetes: a prospective cohort study. J Gen Intern Med.

[28] Egede LE, Nietert PJ, Zheng D (2005). Depression and all-cause and coronary heart disease mortality among adults with and without diabetes. Diabetes Care.

[29] Egede LE, Zheng D (2003). Independent factors associated with major depressive disorder in a national sample of individuals with diabetes. Diabetes Care.

[30] Cassidy F, Ahearn E, Carroll BJ (1999). Elevated frequency of diabetes mellitus in hospitalized manic-depressive patients. Am J Psychiatry.

[31] Lin EH, Von Korff M, Alonso J (2008). Mental disorders among persons with diabetes--results from the World Mental Health Surveys. J Psychosom Res.

[32] de Groot M, Anderson R, Freedland KE, Clouse RE, Lustman PJ (2001). Association of depression and diabetes complications: a meta-analysis. Psychosom Med.

[33] Katon WJ, Rutter C, Simon G (2005). The association of comorbid depression with mortality in patients with type 2 diabetes. Diabetes Care.

[34] Egede LE, Zheng D, Simpson K (2002). Comorbid depression is associated with increased health care use and expenditures in individuals with diabetes. Diabetes Care.

[35] Mezuk B, Eaton WW, Albrecht S, Golden SH (2008). Depression and type 2 diabetes over the lifespan: a meta-analysis. Diabetes Care.

[36] Campayo A, de Jonge P, Roy JF (2010). Depressive disorder and incident diabetes mellitus: the effect of characteristics of depression. Am J Psychiatry.

[37] Sernyak MJ, Leslie DL, Alarcon RD, Losonczy MF, Rosenheck R (2002). Association of diabetes mellitus with use of atypical neuroleptics in the treatment of schizophrenia. Am J Psychiatry.

[38] Huang CJ, Chiu HC, Lee MH, Wang SY (2011). Prevalence and incidence of anxiety disorders in diabetic patients: a national population-based cohort study. Gen Hosp Psychiatry.

[39] Engum A (2007). The role of depression and anxiety in onset of diabetes in a large population-based study. J Psychosom Res.

[40] Hermanns N, Kulzer B, Krichbaum M, Kubiak T, Haak T (2005). Affective and anxiety disorders in a German sample of diabetic patients: prevalence, comorbidity and risk factors. Diabet Med.

[41] Grigsby AB, Anderson RJ, Freedland KE, Clouse RE, Lustman PJ (2002). Prevalence of anxiety in adults with diabetes: a systematic review. J Psychosom Res.

[42] Green L, Feher M, Catalan J (2000). Fears and phobias in people with diabetes. Diabetes Metab Res Rev.

[43] (2004). ‘Schizophrenia and Diabetes 2003’ Expert Consensus Meeting, Dublin, 3-4 October 2003: consensus summary. Br J Psychiatry Suppl.

[44] Lamberti JS, Crilly JF, Maharaj K (2004). Prevalence of diabetes mellitus among outpatients with severe mental disorders receiving atypical antipsychotic drugs. J Clin Psychiatry.

[45] Vinogradova Y, Coupland C, Hippisley-Cox J, Whyte S, Penny C (2010). Effects of severe mental illness on survival of people with diabetes. Br J Psychiatry.

